# Advances in the Role of Dark Septate Endophytes in the Plant Resistance to Abiotic and Biotic Stresses

**DOI:** 10.3390/jof7110939

**Published:** 2021-11-04

**Authors:** Mila Santos, Ignacio Cesanelli, Fernando Diánez, Brenda Sánchez-Montesinos, Alejandro Moreno-Gavíra

**Affiliations:** Departamento de Agronomía, Escuela Superior de Ingeniería, Universidad de Almería, 04120 Almería, Spain; icesanellifilas@gmail.com (I.C.); fdianez@ual.es (F.D.); brensam@hotmail.com (B.S.-M.); alejanmoga@gmail.com (A.M.-G.)

**Keywords:** DSE, endophyte fungi, biological control, abiotic, biotic, stress

## Abstract

Endophytic fungi have been studied in recent decades to understand how they interact with their hosts, the types of relationships they establish, and the potential effects of this interaction. Dark septate endophytes (DSE) are isolated from healthy plants and form melanised structures in the roots, including inter- and intracellular hyphae and microsclerotia, causing low host specificity and covering a wide geographic range. Many studies have revealed beneficial relationships between DSE and their hosts, such as enhanced plant growth, nutrient uptake, and resistance to biotic and abiotic stress. Furthermore, in recent decades, studies have revealed the ability of DSE to mitigate the negative effects of crop diseases, thereby highlighting DSE as potential biocontrol agents of plant diseases (BCAs). Given the importance of these fungi in nature, this article is a review of the role of DSE as BCAs. The findings of increasing numbers of studies on these fungi and their relationships with their plant hosts are also discussed to enable their use as a tool for the integrated management of crop diseases and pests.

## 1. Introduction

Crop production has always been threatened by biotic and abiotic stress. As a result, different tools have been used, from chemical pesticides to genetically modified organisms (GMO), grafts, and physical barriers, to alleviate their impact. As consumers continue to demand products with no agrochemical residues, such as ecological and organic products, some of the traditional methods of control cannot be employed. Biological pest control is thus a current challenge and microorganisms serve as one of the most important tools to enable this control.

Studies on endophytic fungi have been carried out in the last decades to understand how they interact with their hosts, the types of relationships they establish, and the potential effects of this interaction. The main characteristic of endophyte fungi is that they reside and grow within plant tissues, and sporulate during host senescence [1,2,3].

Two major groups of endophytic fungi were previously identified [4]: the clavicipitaceous endophytes (C-endophytes), also known as class 1 endophytes; and the non-clavicipitaceous endophytes (NC-endophytes), which are divided into class 2, 3, and 4 endophytes. C-endophytes are employed to infect grasses, while NC-endophytes are found in asymptomatic tissues of non-vascular plants, ferns, allies, conifers, and angiosperms [4]. Unlike C-endophytes, NC-endophytes have been found in highly diverse types of ecosystems [5].

**Figure 1:**
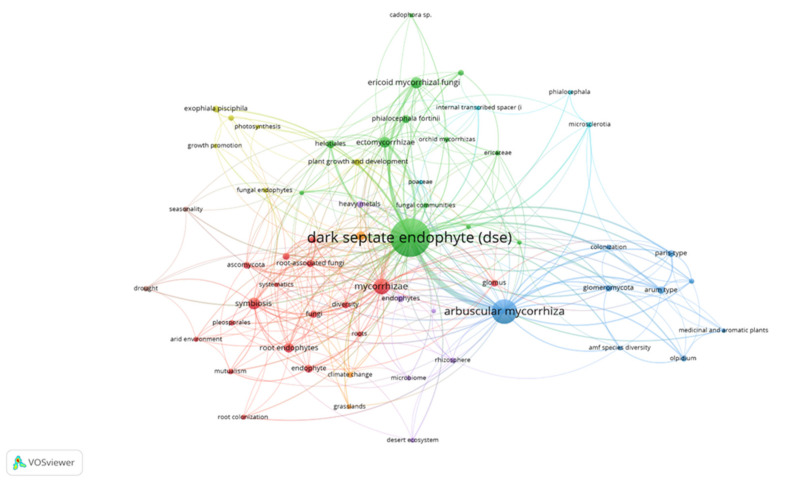
Network map of the co-occurrence matrix for the 547 documents published in DSE research. VOSviewer software (version 1.6.15, Leiden University, The Netherlands) was used to map the frequency of keyword co-occurrence networks. Differences in font size imply differences in relevance. The different colours refer to the groups or clusters formed.

As mentioned above, NC-endophytes can be divided into three classes, and exist in several hosts. The first difference among NC-endophytes is that class 3 endophytes present highly localised infections, while class 2 and 4 endophytes can extensively colonise host tissues. Another difference among the classes is the host tissue that they can colonise. Class 2 can colonise the root, shoot, and rhizome; class 3 can only colonise the shoot; and class 4 colonises only the root of their host [4]. In this review, we will focus on dark septate endophytes (DSE), belonging to the class 4 endophytes. DSE are conidial or sterile septate fungal endophytes, usually isolated from healthy plants, that form melanised structures including inter- and intracellular hyphae and microsclerotia in the roots [4]. DSEs do not present any host specificity as they have been associated with 600 plant species, 320 genera, and 114 families [6,7,8], and can live in a facultative manner on different types of organic compounds [9]. However, certain groups of species show certain preferences regarding attachment to their host [7]. DSE exist over a large geographical range [6,8], from South African coastal plains to tropical, temperate, subalpine, alpine, and maritime Antarctic and Arctic zones [6,10,11]. These ecosystems are usually associated with stressful abiotic conditions, such as Arctic or high-altitude ecosystems [10,11], arid or semiarid regions [12,13], drought, high salt content, low fertility, high CO2 concentration or even heavy metal-contaminated soils [14,15,16,17,18,19,20,21]. The presence of DSE in these types of conditions implies that the DSE-host interaction is crucial to plant survival [19]. This ability can be attributed to the development of melanised hyphae and microsclerotia production [22,23]. The general hypothesis suggested by many studies is that the high concentration of this pigment protects plants from free radicals or heavy metal ions owing to its antioxidant effect [24,25]. However, Graber et al. [26] revealed that salt stress tolerance is independent of melanin accumulation. In other types of studies, melanin content was not found to affect processes such as Agrobacterium tumefaciens-mediated transformation efficiency [27]. The colonization of hosts by DSE follows a similar pattern and begins with the appearance of a certain number of superficial hyphae [28]. The hyphae penetrate spaces between adjacent epidermal cells, continuing to the main axis of the root, and passing through the intercellular spaces of the cortical cells [6,29]. Once the hyphae are inside the root, they form masses of packed pigmented fungal cells within the cortical cells, resulting in microsclerotia development [28]. DSE have been characterised in many studies; however, many of the described species do not have a common phylogenetic group [30,31]. Some DSE species have been included in three groups of Heliotiales, while others have been included in Pleosporales, Sordariales or Pezizales. Most DSE belong to the genera *Cadophora*, *Microdochium*, *Trichocladium* and *Phialocephala* [32]. The *Phialocephala* genus includes species closely related phylogenetically, are indistinguishable, and are grouped in the complex named *Phialocephala fortinii* s.l. (sensu lato)–*Acephala applanata* species complex (PAC). The most frequent DSE in natural forest ecosystems in the Northern hemisphere belong to the PAC [31,33,34,35,36]. *Phialocephala fortinii* was first described in association with *Pinus sylvestris* by Wang and Wilcox [37]. Since then, *Phialocephala fortinii* has been found in many different plant species roots with numerous benefits for the host. Another genus of interest that has been extensively studied is *Exophiala*, which was investigated by Addy et al. [30,34]. This genus is reported to be an endophyte, and can be found in soil, water, and different hosts [38,39,40,41,42]. Recently, different species of *Exophiala* were identified as parasites of the eggs of nematodes of species such as *Heterodera schachtii* [43] or *Tylenchulus semipenetrans* [44]. The genus *Cadophora* includes pathogenic plant species associated with roots or wood colonisers [45]. Wang and Wilcox et al. [37] isolated the *Cadophora finlandica* strain from *Pinus sylvestris* roots. To date, many other species have been characterised; however, their roles in the interactions with their hosts are mainly unknown [46,47]. Beneficial associations between DSE and plants include nutrient uptake [48,49,50,51,52,53,54], growth promoting effects [54,55,56,57,58,59,60,61,62,63], tolerance to abiotic stress, such as heavy metals [64,65,66,67,68], and tolerance to drought [13,19,21,22,57,58,61,69,70,71,72,73,74,75], salinity [27,76,77], biotic stress [78,79,80,81], and changes in the rhizosphere microbiome [73,81,82], thereby positioning DSE as potential tools for crop production and achieving biological control. According to the map shown in Figure 1, there are few species of DSE responsible for the biological control of diseases.

## 2. Nutrient Uptake and Plant Growth Promotion

Nutrient uptake and plant growth are two parameters that are positively correlated. Most studies on DSE–plant interactions could not reveal positive or negative effects [83]; however, some DSE can increase nutritional availability for plants, enabling generally higher growth rates in plants. The effect of the interaction between DSE and the host plant apparently depends on different factors, such as plant and fungal genotype as well as soil fertility [84,85,86,87,88,89]. The enhancement of nutrient absorption by plants has been revealed by numerous researchers. In fact, Xu et al. [52] revealed that the inoculation of maize plants with *E. Pisciphila* H93 enhanced phosphorus absorption by the host plant. This DSE–plant interaction seemed to occur independently of the DSE-induced genes involved in phosphorus absorption [52]. In addition, Vergara et al. [50] reported that DSE stimulated nutrient uptake in rice plants, increasing the accumulation of N, P, K, Mg, Fe, Ca, and Zn in the aerial parts of rice plants. According to He et al. [69], inoculation with DSE increased the content of N and P in plants but decreased the content of organic material and P in the rhizosphere [14,57]. Similarly, Haselwandter and Read [90] reported increased P content in *Carex* plants associated with different species of DSE. Recently, Wu et al. [54] revealed increased expression levels of genes related to metabolic processes and genetic information processes. Moreover, responses to environmental signals were found to be enriched under DSE colonization.

The ability of DSE to colonise and have beneficial effects on plants can also be dependent on the nutrient’s nature in the soil or substrate [89,91,92]. For instance, Yakti et al. [53] identified different biostimulant effects exerted by *Periconia macrospinosa* and *Cadophora* sp., increasing the root and shoot biomass of tomato plants when organic and inorganic nutrient sources were supplied. When organic nutrient sources were applied, only *Periconia macrospinosa* increased shoot and root biomass in tomato plants. However, both DSEs promoted shoot growth when cultivated with inorganic fertilisers.

Mutualistic symbiosis relationships between DSE and crops have been investigated. Such relationships promote nutrient uptake and plant growth. Andrade-Linares et al. [62] reported the biostimulant effect of three DSEs (DSE 48, DSE49 and *Leptodontidium orchidicola*) on tomato plants; however, the effects were only observed in young plants, with none of the DSE strains affecting plants after 22 weeks of cultivation. On the other hand, Usuki et al. [89] revealed that *Heteroconium chaetospira* forms a mutualistic symbiosis with Chinese cabbage, facilitating the supply of nitrogen in exchange for carbohydrates, resulting in increased plant growth.

Interactions between DSE and other microorganisms have been revealed, mainly with arbuscular mycorrhizal fungi (AMF). In many cases, the interactions may involve a stimulation of DSE fungal development [81] or a reduction in the possible pathogenic effect of DSE on the plant [93,94]. Li et al. [95] assayed the co-occurrence of both AMF and DSE *(P. fortinii)* in epidermal cells of hair roots. Based on their results, the use of Finnish peat moss as a symbiotic fungi inoculum could enable the establishment of symbiotic fungal colonization and promote rooting and vegetative growth in rabbiteye blueberry cuttings. In addition, the observed positive effect was greater when the colonisation rates of DSE and AMF were considerably higher. Recently, Guo et al. [96] revealed that DSE and mycorrhizal infection rate significantly improved the bacterial diversity and fruit yield of blueberry rhizosphere soil. *P. fortinii* has been reported to be a plant growth promoter in *Asparagus officinalis, Brassica oleracea,* and *Spinacia* oleracea [6,59,97,98,99,100].

Other processes also promote plant growth, such as the production of different secondary metabolites, volatile organic compounds, and phytohormones by the hyphae of DSE [101,102].

## 3. Abiotic Stress

According to numerous studies, DSE can contribute to the capacity of plants to tolerate abiotic stress, such as salinity, drought, and heavy metal contamination. Different mechanisms have been recognised to be responsible for such protection, such as melanin content in DSE; however, these mechanisms are still unclear, as they often depend on different DSE–host interactions.

Farias et al. [103] revealed that the inoculation of DSE, *Sordariomycetes sp*-B’2 and *Melanconiella elegans*-21W2, in cowpea plants induced tolerance to salt stress. The inoculated plants had higher values for leaf area and shoot and root dry mass than control plants at the same level of water salinity. N and P leaf content, photosynthesis and stomatal conductance were also higher in the inoculated plants. Root colonization by *Piriformospora indica* was found to increase plant growth and attenuate NaCl-induced lipid peroxidation, metabolic heat efflux, and fatty acid desaturation in leaves of the salt-sensitive barley [104]. *P. indica* significantly elevated the amount of ascorbic acid and increased the activities of antioxidant enzymes in barley roots under salt stress conditions. Other authors revealed that *P. indica* colonization promoted *Arabidopsis* growth under salt stress conditions, which might be caused by modulation of the expression levels of the major Na^+^ and K^+^ ion channels [105].

Hou et al. [72] revealed that the symbiotic effect of DSE on *Artemisia ordosica* depended on the DSE species and the salt concentration of the medium. Accordingly, *Alternaria chlamydosporigena* was found to promote the accumulation of total biomass and enhance superoxide dismutase (SOD) activity; *Paraphoma chrysanthemicola* promoted the accumulation of root biomass and increased indoleacetic acid (IAA) contents; and *Bipolaris sorokiniana* enhanced SOD activity and glutathione (GSH) and IAA contents, with changes in the rhizosphere microbiome depending on the salt concentration. Conversely, Gonçalves et al. [106] found that the beneficial effect of the mutualistic interaction between *Salicornia* and *Stemphylium* sp. decreased as the salt concentration increased.

Drought is another abiotic stress where the positive effect of DSE was revealed. The interaction between DSE and their hosts is apparently stronger under water stress conditions. Accordingly, the interaction between *P. indica* and water stress was found to activate signal oxidation during stress to prevent cell damage and assist in the maintenance of the osmotic pressure in artichoke (*Cynara scolymus*). Thus, higher water stress levels in the host plant might be attributed to the increased content of phenolics and flavonoids [107]. On the other hand, Li et al. [75] found that *Phialophora* sp. and *Leptosphaeria* sp. improved the root biomass, total biomass, nutrient concentration, and antioxidant enzyme activities of host plants under water deficit conditions. The same results were found in cases of colonisation by the DSE fungal group known as PAC (*Phialocephala fortinii* s.l.–*Acephala applanata* species complex) [35], which increased under water stress conditions. Landold et al. [100] suggested that the PAC could protect oak roots against desiccation and improve the drought resistance of *Q. pubescens* due to the more extensive formation of waterproof microsclerotia. Stroheker et al. [63] also proposed the above notion in their assessment of *P. abies* seedlings. Zhang et al. [108] revealed that sorghum plants inoculated with *Exophiala pisciphila* showed significantly higher growth and dry matter content than non-inoculated plants, alleviating the negative effects of drought. In addition, plants inoculated with *Exophiala pisciphila* had higher photosynthetic and leaf transpiration rates. Liu et al. [70] found that *Acrocalymma vagum* could establish a symbiotic relationship with *Ormosia hosiei*, allowing the hosts to improve their defence against drought stress, reduce water loss, and maintain normal physiological activity. However, the researchers found that inoculated plants had higher contents of chlorophyll a, chlorophyll b, and carotenoids, with increases of 13%, 28%, and 35%, respectively. Inoculation was also found to significantly enhance net photosynthetic rate, stomatal conductance, and transpiration rates. Morphology differences were also found in leaves, with inoculated plants displaying greater leaf length, area, dry weight, and thicker leaves. According to Panke-Buisse et al. [109], reduced numbers of DSE are associated with a greater drought tolerance. However, there are numerous DSE genera found in plants, such as *Paraconiothyrium, Phialophora, Darksidea, Knufia, Leptosphaeria* and *Embellisia*, that lead to drought resistance in other plant species [57,110].

The important role of DSE in heavy metal buffering and tolerance in plants is well documented. Metal resistance to Cd and Zn was described to result from DSE and plant interaction [111,112,113,114,115,116,117]. Zhu et al. [118] revealed that two strains of *Phialophora mustea* improved the tolerance of metal stress and promoted plant growth and dry weight in tomato plants. Similarly, *P. fortinii* could promote the growth and survival of *Miscanthus sinensis* in mine sites under Al stress [119]. This tolerance was based on the reduction in metal contents in roots and shoots and the limited translocation from roots to shoots in inoculated plants compared with non-inoculated controls. DSE stimulated the activities of antioxidant enzymes, superoxide dismutase (SOD), and peroxidase (POD), mitigating the membrane lipid peroxidation damage caused by excessive metal ions. In another study, Shen et al. [120] assessed how maize responds to Cd stress following *Exophiala pisciphila* inoculation and found that the DSE alleviated the toxic effect of Cd excess and promoted plant growth. Furthermore, Cd accumulation in the root cell walls was higher in inoculated plants; thus, inoculated plants showed a decrease in Cd translocation from roots to shoots. Shadmani et al. [113] determined the effect of Cd uptake and growth on barley plants inoculated with *Microdochium bolleyi* under three Cd concentrations. Based on their results, root and shoot growth and dry root and shoot biomass were significantly higher in inoculated plants than control plants under all Cd concentration conditions. The study also revealed that Cd concentration in shoots did not increase under higher levels of Cd in the soil due to an efficient regulation system that minimised the heavy metal concentration in the aerial parts of the plant. Similar results were obtained by Yung et al. [121] when *Leptodontidium* sp. and *Phialophora mustea* were employed to inoculate *Noccaea caerulescens* plants, which contributed to the phytoextraction of heavy metals in polluted soils and increased plant growth.

Ban et al. [122] sought to determine the effect of different Pb concentrations on maize growth and photosynthesis efficiency when plants were inoculated with *Gaeumannomyces cylindrosporus.* Based on their results, inoculated plants had higher height, basal diameter, root length and total biomass, and had more efficient photosynthesis parameters than control plants. Similar to other cases, the results revealed that inoculated plants accumulated more Pb than control plants, and this accumulation was restricted to the roots. This finding suggests that in the inoculated plants, the translocation patterns were changed. Consequently, Pb accumulation in the roots alleviated the aerial parts of the plants.

The use of DSE fungi in phytoremediation can help to minimise the effect of high TE-polluted soils on plants [123]. Previously, Berthelot et al. [124] identified different DSE species isolated from the roots of poplar trees that could produce VOCs and phytohormones, and promote the development of *Betula pendula*, *Eucalyptus globulus* and *Lolium perenne*. Similarly, *Exserohilum pedicellatum, Ophiosphaerella* sp. and *Alternaria alternata* displayed technological potential for application in phytoremediation processes owing to their ability to degrade oil [125]. DSE can also contribute to a reduction in the damage caused by global warming in different ecosystems, individually or in combination with other microorganisms [126].

## 4. Biotic Stress: Biological Control

### 4.1. Mechanisms of Action

**Figure 2:**
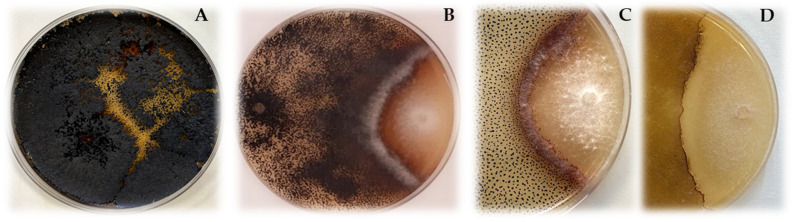
(**A**). Microsclerotia formed by DSE *Rutstroemia calopus*. (**B**). Inhibitory effect of DSE *R. calopus* against *F. solani* on PDA medium. Effect of salt stress (**C**): PDA amended with 5 g·L^−1^ of NaCl; (**D**): PDA amended with 20 g·L^−1^ of NaCl) on microbial antagonism of *R. calopus* against *Sclerotinia sclerotiorum*. High salt concentrations significantly reduced the number of microsclerotia and melanin accumulation. No reduction in the antagonistic effect of DSE was observed.

Several researchers evaluated the role of DSE as biocontrol agents of plant diseases, reporting positive results via in vitro and in vivo assays. Fungal strains can establish different types of interactions, such as competition for space, nutrients (Figure 2), or antibiosis, or the development of plant resistance to phytopathogens. Secondary metabolites, such as siderophores or acid compounds, can mobilise nutrients in favour of one strain, thereby limiting the nutritional resources to the other. Secondary metabolites can also reduce growth on the other fungus, generating an antibiosis response [127]. DSE-induced resistance to plant diseases has also been described [128].

Interactions between DSE-hosts and biological control mechanisms have been reported in many studies. For instance, Tellenbach et al. [78] studied the mechanism employed by *Phialocephala* strains to control *Phytophthora* species. The *P. europaea* strain significantly reduced *Phytophthora plurivora* growth compared with the other strains evaluated. During the evaluation of metabolite production, four compounds were found: sclerin, sclerolide, sclerotinin A, and sclerotinin B. Iron is an essential micronutrient for every organism. Bartholdy et al. [129] tested the capacity of *Phialocephala fortinii* strains to excrete siderophores into the nutrient medium for the mobilization and uptake of nutrients, limiting the resource for other fungi. In the assay, the researchers identified ferricrocin, ferrirubin, and ferrichrome C as the main types of excretions. Siderophore production is influenced by several factors. For example, the researchers measured the highest siderophore concentrations at low pH values (4–4.5), whereas the highest levels of pH tended to inhibit siderophore excretion. The initial Fe concentration also affected siderophore synthesis. Although some initial Fe concentration is required for growth, high iron concentration inhibited siderophore synthesis. Thus, optimal levels depend on each individual organism.

Su et al. [130] found that *Harpophora oryzae* could control *Magnaporthe oryzae* in rice plants by inducing the accumulation of H_2_O_2_, an important type of ROS, in the site of infection; this effect was proportional to the stage of development of the DSE. In addition, the presence of ROS in the non-infected cells adjacent to the primary infection could be observed, inducing resistance to secondary infections. When foliar infection was analysed, it was clear that unlike susceptible control, plants inoculated with *H. oryzae* limited the lesions of *M. oryzae* to localised spots, controlling the damage, and indicated that *H. oryzae* induced systemic resistance to the disease. Deng et al. [79] studied how *Phialocephala bamuru* and *Rhizoctonia solani* affect physiological indices in *Pinus Sylvestris var. mongolica*. By comparing *P. bamuru* and control seedlings inoculated with *R. solani*, the researchers found that β–1,3-Glucanase and chitinase activity, proline, and soluble protein contents in seedlings from treatment via DSE inoculation increased by 18.41%, 92.54%, 101.27%, and 30.48%, respectively, in comparison to the control. In the study, the survival rate significantly increased from 33% with control treatment to 80% with *P. bamuru* treatment.

Understanding the mechanisms of action of the different DSE that can control diseases, as well as the different interactions that occur between DSE, the host and other microorganisms present in the rhizosphere of the plant, is of great importance for its potential use in agriculture. It is important to unravel not only the advantages, but also the disadvantages, such as potential risk of toxicity to the environment or human health. From a commercial perspective, the high microsclerotia formation (Figure 2A) rate of DSE can lead to higher viability of the future formulation. In addition, it is important to understand whether any additional requirements are necessary to increase the benefit of DSE in the plant, or if, on the contrary, the nutrient solution and/or water requirements can be adjusted without any loss in production.

### 4.2. Biological Control Assays

The role of DSE in the biological control of plant diseases has been less studied than their effects as biostimulants under abiotic stress conditions. Nevertheless, several studies have reported that DSE fungi can be used as biocontrol agents for pathogenic fungi (Table 1). The beneficial effects of their application in plants include changes in root architecture, disease alleviation, and improved plant growth, among other benefits.

Vascular diseases caused by *Fusarium* and *Verticillium* are controlled using different DSE species. Khastini et al. [131] studied the control of *Fusarium* wilt by applying *Cadophora* sp. to melon seedlings in petri dish and field tests. The results revealed disease alleviation of 40–65% in both types of tests, with greater hyphal colonisation in the roots when the amino acid valine was added to the medium.

Appositions and thickenings were observed in the cells of the plant cell wall. Similar results were found in a biocontrol assay of *Veronaeopsis simplex* against *Fusarium oxysporum* in Chinese cabbage [142], which resulted in 70% disease alleviation. Competition for space or infection sites between *V. simplex* and *F. oxysporum* was observed; however, no direct antagonistic activity was demonstrated in vitro. On the other hand, Surono [141] detected 100% disease alleviation caused by *Fusarium oxysporum* f. sp. *asparagi* by applying *Phialocephala fortinii.* Harsonowati et al. [133] reported the control of *Fusarium oxysporum* f. sp. *fragariae* by *Exophiala* sp., *Exophiala pisciphila*, and *Cladophialophora chaetospira*, each causing disease alleviation of 62, 85, and 90%, respectively. In addition, DSE promoted plant growth by increasing photosynthetic rates and accelerating flower initiation and fruit formation.

Andrade-Linares et al. [62] reported the control of *Verticillium dahliae* in tomato plant assays using DSE49 and *Leptodontidium orchidicola,* which reduced the severity of the disease by 30% under field conditions. Narisawa et al. [80] also evaluated the interaction between *Phialocephala fortinii* and *Heteroconium chaetospira* against *Verticillium longisporum* in Chinese cabbage under field conditions and found a reduction in the disease by 80% and 50%, respectively. Other important pathogens have also been studied. Berthelot et al. [127] evaluated the interaction between DSE and pathogen in dual cultures, and reported that the growth of the pathogen, *Heterobasidion annosum*, was inhibited by *Phialophora mustea* and *Cadophora* sp. Moreover, *P. mustea* was found to inhibit *Phytophthora citricola*. On the other hand, Berthelot et al. [127] employed DSE/DSE strains but did not find any negative effects in the interaction. Finally, Tellenbach and Sieber [143] demonstrated that *Phialocephala subalpina* could reduce disease intensity caused by *Elongisporangium undulatum* and *Phytophthora plurivora* in Norway spruce seedlings.

As previously discussed, the parasitism of nematode eggs and juveniles by DSE has been revealed [43,44,134,135]. Gené et al. [44] found that parasitism was related to the presence of eggs or females and to the amount of magnesium or phosphorus in the soil. Disease alleviation by DSE was related to changes in root architecture as well as changes and conservation of the microbiota present in the rhizosphere. However, to our knowledge, very few studies have examined the control of bacterial diseases by DSE [128].

DSE do not always suppress pathogens. For instance, Yakti et al. [132] studied the in vitro interaction between *Cadophora* sp and three pathogens, namely *Verticillium dahliae, Rhizoctonia solani*, and *Pythium aphanidermatum.* Based on their findings, although *Cadophora* sp. reduced the growth of pathogens, the pathogens also reduced the growth of DSE at a higher percentage. As this behaviour was not observed in plant tests, the in vitro results were not consistent with the in vivo results. Similarly, Martínez-Arias et al. [138] did not obtain good results for control of *Ophiostoma ulmi* in *Ulmus* by applying *Exophiala* sp. However, studies revealed the inhibition of the pathogen, but to the detriment of plant growth [144]. In general, the interactions between DSE and their hosts are not necessarily beneficial. Although DSE do not behave as a pathogen, they can lead to a reduction in plant growth [144].

According to Berthelot et al. [102], studies to date are still at an early stage and thus require more mature validation. New DSE with different functions are being characterised [145], such as the production of anticancer metabolites which, however, showed no biostimulant effect in the tested plant species [146], or biostimulants of growth and production of anticancer compounds in plants [147]. More studies are required to understand the different interactions of DSE within cultivation systems and to incorporate DSE into pest and disease management methods. Compatibility studies with fungicides commonly used in agriculture are also needed [148].

## 5. Conclusions

Although more information has been obtained concerning the role of DSE in alleviating biotic stress, much work remains to be done before DSE can be successfully introduced into the market. Market trends and legislation for agricultural production systems highlight the need for products that are environmentally friendly and waste-free and are produced under environmentally sustainable parameters. Consequently, workers in the agricultural sector must follow these trends and develop production models that meet these requirements. As part of this adaptation, alternatives to disease control that eliminate the use of chemical tools as much as possible should be discovered and employed. As revealed in this review, DSE can colonise and establish relationships with their hosts, resulting in benefits to plants not only in terms of disease control, but also in terms of growth, nutrient solubilisation and absorption, and tolerance to abiotic stresses, such as salinity and drought. Accordingly, we believe that these types of fungi can become a very important player in new agricultural production methods that will be presented in the short and medium term. Therefore, their behaviour within these methods must be studied. Agronomic studies are necessary to determine how the DSE present in each ecosystem can be incorporated into the current production systems of each region and crop, integrating them as tools for daily use.

## Figures and Tables

**Table 1:** In vitro and in vivo biological control tests carried out using DSE.

DSE	Phytopathogen	Host	References
*Cadophora* sp.	*Fusarium oxysporum *f. sp.* meloni*	*Cucumis melo*	[131]
	*Verticillium dahliae*	In vitro/tomato	[132]
	*Rhizoctonia solani*	In vitro/tomato	[132]
	*Pythium aphanidermatum*	In vitro/tomato	[132]
	*Heterobasidion annosum*	In vitro	[127]
*Cladophialophora chaetospira*	*Fusarium oxysporum*	*Brassica pekinensis*	[133]
*Cladosporium cladosporioides*	*Bursaphelenchus xylophilus*	*Pinus tabulaeformis*	[134,135]
		*Fragaria vesca*	[133]
*Cryptosporiopsis ericae*	*Heterobasidion* *parviporum*	In vitro	[136]
*Cryptosporiopsis* sp.	*Phytophtora pini, Heterobasidion parviporum*	In vitro	[137]
	*Botrytis cinerea*		
*Exophiala pisciphila*	*Fusarium oxysporum*	*Brassica pekinensis*	[133]
	*Fusarium oxysporum *f. sp.* fragariae*	*Fragaria vesca*	[134]
	*Tylenchulus semipenetrans*	Screening/in vitro	[44]
*Exophiala salmonis*	*Bursaphelenchus xylophilus*	*Pinus tabulaeformis*	[134]
*Exophiala sp.*	*Fusarium oxysporum*	*Brassica pekinensis*	[133]
	*Fusarium oxysporum *f. sp.* fragariae*	*Fragaria vesca*	[133]
	*Heterodera schachti*	*Beta vulgaris*	[43]
	*Ophiostoma ulmi*	Ulmus	[138]
*Gaeumannomyces cylindrosporus*	*Bursaphelenchus xylophilus*	*Pinus* spp.	[135]
	*Bursaphelenchus xylophilus*	*Pinus tabulaeformis*	[134]
*Harpophora oryzae*	*Magnaporthe oryzae*	Rice	[130]
*Heteroconium chaetospira*	*Verticillium longisporum, Plasmodiophora brassicae*	*Brassica pekinensis*	[80,139]
	*Pseudomonas syringae pv. Macricola, A. brassicae*		[128]
*Leptodontidium orchidicola*	*Verticillium dahliae*	Tomato	[62]
*Leptodontidium* sp	*Pythium intermedium, Phytophthora citricola, H. annosum*	In vitro	[127]
*Meliniomyces variabilis*	*Verticillium longisporum*	*Brassica pekinensis*	[80,140]
*Paraphoma chrysanthemicola*	*Bursaphelenchus xylophilus*	*Pinus* spp.	[135]
		*Pinus tabulaeformis*	[134]
*Phialocephala bamuru*	*Rhizoctonia solani*	*Pinus silvestris*	[79]
*Phialocephala sphareoides*	*Heterobasidion* *parviporum*	In vitro/*Picea abies*	[134]
*Phialocephala europaea*	*Phytophthora citrícola*	In vitro	[127]
*Phialocephala fortinii*	*Verticillium longisporum*	*Brassica pekinensis*	[80]
	*Fusarium oxysporum *f. sp.* asparagi*	*Asparagus officinalis*	[141]
*PAC*	*Heterobasidion* *parviporum*	In vitro	[136]
*Phialophora mustea*	*Phytophtora citricola, Pythium intermedium H. annosum*	In vitro	[127]
	*Bursaphelenchus xylophilus*	*Pinus tabulaeformis*	[134]
*Phialocephala subalpina*	*Phytophthora plurivora*	In vitro	[132]
	*Elongisporangium undulatum*	In vitro	[132]
*Veronaeopsis simplex*	*Fusarium oxysporum*	In vitro	[142]
